# Tegner level is predictive for successful return to sport 2 years after anterior cruciate ligament reconstruction

**DOI:** 10.1007/s00167-020-06335-4

**Published:** 2020-10-28

**Authors:** Antonio Klasan, Sven Edward Putnis, Samuel Grasso, Vikram Kandhari, Takeshi Oshima, David Anthony Parker

**Affiliations:** 1 Sydney Orthopaedic Research Institute, 445 Victoria Ave, Chatswood, NSW 2067 Australia; 2 Department for Orthopaedics and Traumatology, Kepler University Hospital GmbH, Johannes Kepler University Linz, Krankenhausstrasse 9, 4020 Linz and Altenberger Strasse 69, 4040 Linz, Austria

**Keywords:** ACL reconstruction, Return to sports, Rehabilitation, Tegner

## Abstract

**Purpose:**

For a successful return to sport (RTS) after an anterior cruciate ligament reconstruction (ACLR), patients are recommended to attend a comprehensive rehabilitation program, followed by an RTS assessment, that is a combination of tests. The purpose of this study was to predict a successful return to sport using the results of the RTS assessment and self-reported questionnaires at minimum 2 years after ACLR.

**Methods:**

A total of 123 consecutive ACLR patients undertook an intensive rehabilitation program followed by a comprehensive RTS assessment that included an established combination of balance and strength tests, the ACL-return to sport after Injury scale (ACL-RSI) questionnaire and a KT1000 laximetry test. Preinjury and expected Tegner and Lysholm were collected at baseline, at RTS and prospectively collected at minimum 2-year follow-up. The patients were asked if they returned to their previous sport and at which level. All variables were included in a regression analysis predicting a successful return to previous sport, return to the same level of sport as well as the Tegner level at 2 years.

**Results:**

Sixty-two patients (50%) returned to their previous sport by the 2-year follow-up, without a difference in preinjury Tegner between these two groups (n.s.). Expected preoperative Tegner was the only significant predictor of a successful return to previous sport (*p* = 0.042; OR 1.300, 95% CI 1.010–1.672). Out of the 62 patients returning to their previous sport, 38 (61%) reported to be on the same or higher level. The only predictive variable for returning to the same level was the higher preinjury Tegner level (*p* = 0.048; OR 1.522). Multivariate regression analysis of Tegner level at 2 years found younger age to be the only predictive value. From the RTS assessment tests, the ACL-RSI questionnaire and the posterolateral balance test were predictive variables for Tegner at 2-year follow-up, albeit in the univariate regression analysis.

**Conclusions:**

Preoperative Tegner and expected Tegner level collected prior to an ACL reconstruction can aid in the objective prediction of patients’ return to sport after 2 years. High-level athletes are more likely to return to their previous sport and to the previous level. Younger patients achieve a higher Tegner level at 2 years.

**Level of evidence:**

Level III study.

**Electronic supplementary material:**

The online version of this article (10.1007/s00167-020-06335-4) contains supplementary material, which is available to authorized users.

## Inroduction

Anterior cruciate ligament (ACL) rupture is one of the most common sports injuries with more than 250,000 injuries and 125,000 reconstructions occurring yearly in the United States alone [9]. There is limited evidence to support nonsurgical management even for less active patients with less laxity [36]. Patients undergoing operative treatment should undergo a comprehensive rehabilitation program, regardless of their intent to return to any sports activity [25]. In spite of advanced surgical techniques and comprehensive rehabilitation programmes, the risk of graft rupture is still substantially high, ranging from 4% [5] up to 20% [20].

Return to sport (RTS) assessments have been introduced to advise patients about timing of safer return to sport, and reduce the risk of graft rupture, by performing a series of qualitative and quantitative measurements of strength and balance [25]. Achieving a limb symmetry index of  > 90% has been shown to be beneficial in preventing graft rupture [19]. However, safe return to the same pre-injury sporting level is still a widely discussed debate, with a growing body of evidence and more than 300 papers published on this topic just between 2014 and 2017 [7]. Even though recommendations for safe return to sport include both time-based and performance-based criteria, a recent systematic review found that less than one in five of the studies combine these two factors [33].

A further complexity is defining return to sports [8]. It can range from retuning to any sport to return to previous sport and previous level of participation. There are sport-specific differences in RTS as well which is biasing the literature even further [26]. Finally, the transfer of knowledge to practical routine seems suboptimal [21], which could be a consequence of the factors mentioned above. Only 10% of level I and level II studies report if patients were able to return to sports and 75% do not report the level they returned to [8].

The aim of this study was to analyse the results of an RTS assessment and the patient-reported questionnaires, its elements and to predict a successful return to sport, including the Tegner level. We hypothesised that the preoperative Tegner level can predict a successful return to sport and that the elements of the RTS assessment cannot predict a successful return to sport. It was hypothesised that preoperative Tegner will be predictive of the Tegner level at 2 years.

## Materials and methods

### Patient selection and surgical data

This is a retrospective study of consecutive patients approved by our institutional ethics committee (Northern Sydney Local Health District; HREC/17/HAWKE/140). Recruited participants underwent a primary arthroscopic ACL reconstruction by one of three senior orthopaedic surgeons using hamstring autograft. The tibial end of the hamstring autograft was fixed with suspensory fixation in 82.6% of the cases and with a non-resorbable interference screw and sheath in 14.4% of the cases, whereas the femoral end was fixed using adjustable suspensory fixation in all cases. Graft size was measured intraoperatively using the tightest fit in a grooved sizing block which also corresponded to the tunnel width. The procedures were performed either as a single overnight stay or as outpatient procedures. After discharge, rehabilitation was supervised by a patient-selected physiotherapist approved by the treating surgeon. Treating physiotherapists were given the same rehabilitation protocol guidelines (Supplement 1). The rehabilitation protocol focused on early recovery of full active knee extension and quadriceps function. Weightbearing was permitted as tolerated and range of motion was unlimited from the first postoperative day.

The inclusion period was between August 2015 and March 2017. Included were patients older than 18 years, without limitations on gender, BMI or type of sport. Patients with meniscal and chondral injuries were included. Exclusion criteria were patients with previous knee ligament injuries, patients with multi-ligament injuries and patients who did not intend to return to sport after their ACL reconstruction.

### Preoperative assessments

After patient consent for ACL reconstruction, but prior to surgery, baseline data were collected. Patient sport activity level was assessed with the Tegner activity level (Tegner) [39]. Patients were also asked to fill out their expected sports activity level after recovery from the ACL reconstruction, again using the Tegner [39]. To assess the knee function, Lysholm knee scale [39] and International Knee Documentation Committee (IKDC) Scale [14] were collected. Side-to-side knee laxity was assessed using the KT1000 (MEDMetric Corporation, San Diego, CA, U.S.) preoperatively on the healthy and ACL deficient leg with the force of 134 N and a manual maximum force in a standardised manner [3], by a total of four investigators, of whom each has performed at least 50 measurements prior to study commencement.

### Return to sport assessment

At 9 months postoperatively, patients underwent a comprehensive RTS assessment. Eligibility for the assessment was clearance by the surgeon and the following criteria: no effusion, full range of motion, a minimum of 8 weeks unrestricted training and the clinical examination demonstrating a stable graft, using the Lachman Test [35]. Follow-up Tegner, Lysholm and IKDC scores were collected. Patients’ height and weight were documented, and patient’s range of motion of both knees was measured with a goniometer and documented. Psychological readiness for returning to sport was assessed using the Anterior Cruciate Ligament-Return to Sports After Injury (ACL-RSI) (short version) scale [43]. Knee laxity measurements were performed using the KT1000, again at 134 N and at manual maximum force on both legs in the same manner [3], measured in millimetres. The battery of tests used in this study adhered to the latest best practice recommendations, which state that at least a strength test battery and a hop test battery as well as quality of movement measurement used for determining the moment for safe return to sports [25].

Star excursion balance test was performed using the Y Balance Test (YBT) Kit™ [32] (Perform Better, West Warwick, RI, USA). The maximal reach distance was measured by reading the tape measure at the edge of the reach indicator, at the point where the most distal part of the foot reached, recorded in cm. The testing order was: anterior, posteromedial and posterolateral for the uninjured leg, followed by the same order for the injured leg. Patients are allowed three practice trials followed by three recorded trials [15]. The scores from the best trials were used as the final results. In case the final trial yielded the best result, the patients are allowed subsequent trials until they achieve the highest result.

The following single-leg hop tests were used: single hop for distance [31], single hop for height [40] and timed repetitive side hop [11]. All tests were performed in the same order, uninjured leg first followed by the injured leg. For the distance and height tests, a total of 6 trials were allowed and recorded, with the best result recorded. Hop height was calculated using the time (t) in the air, captured by a slow motion camera and calculated using the formula ½*g*(*t*/2)^2^; (where *g* = 9.81 m/s^2^), recorded in tenths of a second [27]. For the side hops, the subjects stood on the test leg, and jumped from side-to-side between two parallel strips of tape, placed 40 cm apart on the floor. The subjects were instructed to jump as many times as possible during a period of 30 s. The number of successful jumps performed, without touching the tape, was recorded and the number of failed attempts was also recorded. The side hop test was allowed only once.

Absolute values were recorded. Side to side difference was calculated by subtracting the injured leg result from the healthy leg result. The limb symmetry index (LSI) was defined as the ratio of the involved limb score and the uninvolved limb score expressed in per cent (involved/uninvolved × 100 = LSI). In this study, an LSI greater than or equal to 90% was considered normal [17]. LSI less than 90% in one of the categories was deemed a pass, LSI of less than 90% in two or more was deemed a failure. Throughout the assessment, verbal encouragement was used and patients wore full sports gear with low, rubber-soled sports shoes. Patients failing the RTS assessment were advised to continue physiotherapy for a period of 12 weeks and redo the RTS test. They were also advised against returning to sport during this time.

### Graft ruptures and final follow-up

Clinical follow-up was at 6 weeks, 3, 6 months and at the time of the RTS assessment. At a minimum of 2 years after surgery, all the patients were followed up. Further, IKDC, Tegner and Lysholm scores were collected via Web-Survey (Socrates, Ortholink, Pyrmont, NSW, Australia) or telephone. The patients were asked if they (a) returned to their previous sport and (b) if they feel they were performing on the same level as prior to the ACL injury.

### Statistical analysis

The success of returning to previous sport and the level of performance were assessed subjectively by asking the Tegner patients if they felt they returned to these levels and objectively using the rating system [12]. It was described in 1985 to, complementing the Lysholm questionnaire, to assess the sporting level of the patient [4]. Binominal logistic regression was used to predict the following events: returning to previous sport and returning to the same level at minimum 2-year follow-up. Linear regression was used to predict the Tegner level at minimum 2-year follow-up. A model was created for each of these analyses. First, a univariate regression analysis was performed with the following variables: age at surgery, gender, femoral graft tunnel size, tibial graft tunnel size, intraoperatively treated meniscal or chondral injury, preinjury sport category, preinjury Tegner level, expected Tegner level, preinjury IKDC score, preinjury Lysholm score, ACL-RSI score, YBT anterior, posteromedial and posterolateral side difference in cm as well as LSI for each measurement; difference in hop distance in centimetre for both legs as well as LSI; difference in hop height in centimetre between legs as well as LSI; number of successful side hops for both legs as well as percentage of failed side hops and finally, KT1000 measurements at 134 N and manual maximum force level with an absolute value for a side-to side difference as well as a threshold of 3-mm side-to side difference [28]. The LSI was considered normal at 90% [34]. After determining statistically significant variables in the univariate regression analysis, a multivariate regression analysis was performed to control for co-variates. Normality of data was tested with the Shapiro–Wilk test. Non-normally distributed continuous variables are shown as median (range) and were compared using the Mann–Whitney *U* test, normally distributed continuous variables were compared using the *t* test. Due to observed statistical significance for all primary outcomes in all analyses [16] and the limited number of patients, a formal power analysis was not performed. Statistical significance was set at 5%. Statistical analysis was performed using SPSS 24 (IBM, Armonk, NY, USA).

## Results

**Fig. 1:**
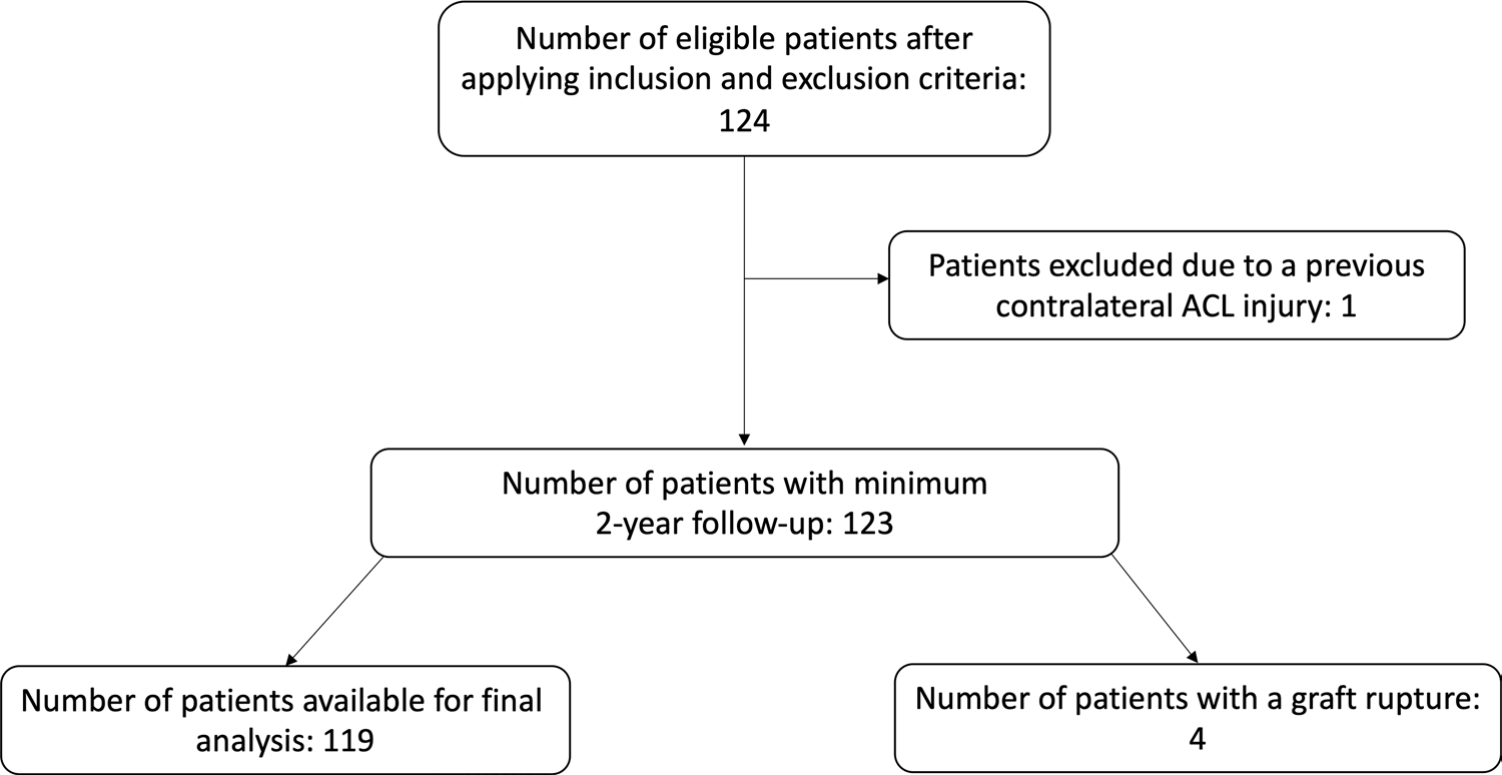
Patient inclusion flow chart

After the application of inclusion and exclusion criteria, 123 patients were eligible for the study (Fig. 1), with the preoperative parameters reported in Table 1. One patient was excluded due to a previous contralateral ACL injury, which potentially could have influenced his Tegner level and subjective level of participation. There were 59 patients (48.0%) with a concomitant meniscal injury and 9 patients (7.3%) with a chondral injury. All patients underwent the RTS assessment and completed follow-up IKDC, Tegner and Lysholm questionnaires. One patient had LSI < 90% in two categories, which meant he failed the RTS assessment. He repeated the return to sport assessment after 12 weeks and subsequently passed, and all other patients passed first time. At minimum 2-year follow-up, four patients had sustained a graft rupture, all occurring within the first 6 months after passing the RTS, giving a graft rupture rate in the overall cohort of 3.2%.

**Table 1:** Patient demographics and characteristics

Parameter	Value
Age, median (range)	30 (18–60)
Female gender %	42.3%
BMI, median (range)	24.6 (18.5–47.9)
Preinjury tegner, median (range)	8 (5–10)
Preoperative IKDC, mean (standard deviation)	48.3 (15.5)
Preoperative lysholm, mean (standard deviation)	64.4 (18.0)
Tegner at 2-year follow-up, median (range)	6 (1–10)
IKDC at 2-year follow-up, mean (standard deviation)	85.0 (10.2)
Lysholm at 2-year follow-up, mean (standard deviation)	91.4 (7.8)
Total cases	123

### Returning to the previous sport

Sixty-two patients (50%) returned to their previous sport by the 2-year follow-up. There was no difference in preinjury Tegner between these two groups (n.s.), but Tegner level was higher in the group that returned to sport at minimum 2-year follow-up (*p* < 0.001). The median IKDC score at minimum 2-year follow-up was 88.5 (63–100) in the group that returned to sport and 82.8 (41–99) in the group not returning to sport (*p* = 0.001), with no difference preoperatively (n.s.). Expected preoperative Tegner was the only significant predictor of a successful return to previous sport in the univariate analysis (*p* = 0.042; OR 1.500, 95% CI 1.010–1.672). Concomitant injuries were not predictive of returning to the previous sport.

### Returning to the same level of previous sport

Out of 62 patients returning to the previous sport, 37 (59%) reported to be on the same level and one reported to be on a higher level. The only predictive variable for returning to the same level was the higher preinjury Tegner level (*p* = 0.048; OR 1.522). Concomitant injuries were not predictive of returning to the same level of previous sport. There was no difference in preoperative Tegner between the group that returned and did not return to sport (n.s.); however, Tegner at the latest follow-up was higher in the group returning to the same level than in the group not achieving the same level (*p* < 0.001).

### Predicting the Tegner level

When predicting the Tegner at minimum 2-year follow-up, after controlling for covariates, lower age was the only predictive variable (Table 2). However, ACL-RSI questionnaire as well as the expected Tegner were significant at univariate analysis. From the RTS battery of tests, posterolateral YBT limb symmetry index was the only predictive variable for Tegner at 2-year follow-up, albeit, of lower predictive value than the questionnaires and the patient age.

**Table 2:** Multivariate linear regression results with Tegner level at minimum 2-year follow-up as the dependant variable

Variable	*p* value	Coefficient	Coefficient 95% confidence interval
Age at surgery	0.001	−0.051	−0.081 to 0.021
ACL-RSI	n.s	0.010	−0.005 to 0.025
Expected tegner	n.s	0.157	−0.070 to 0.384
YBT, PL, LSI	n.s	0.370	−0.004 to 0.079

*ACL-RSI* anterior cruciate ligament-return to sports after injury, *YBT *Y balance test, *PL* posterolateral, *LSI *limb symmetry index

## Discussion

The most important findings of the present study were the expected preoperative Tegner level as a predictor of a successful return to previous sport, and the preinjury Tegner as a predictor of a return to the same level of the previous sport. Linear regression revealed younger age to be predictive of Tegner level at minimum 2-year follow-up in the multivariate analysis. ACL-RSI questionnaire, expected Tegner level and posterolateral Y-balance test limb asymmetry index were predictive on univariate analysis. Our first hypothesis was confirmed, and the second was rebutted.

Return to sport after an ACL injury is complex. A recent systematic review found that 82% of patients after ACL injury return to sport, but only 63% return to their previous sport [1]. The numbers in the present study are lower, which may be due to the inconsistency of reporting rates of return to the same sporting level [6, 8]. This is a major limitation when comparing studies, which was one of the main drivers for an in-depth analysis of not only returning to sport, but also clearly distinguishing between returning to sport and returning to the same sporting level, both subjectively perceived by the patient and captured by the Tegner questionnaire. Comparatively, the majority of RTS components could not predict a return to sport. It is recommended that an RTS assessment includes a strength test battery, a hop test battery as well as quality of movement measurement [25]. The overall result can be used to determine the readiness to return to sport, but the individual components cannot predict the level of return, as was demonstrated in the present study. This lack of predictive value of the RTS tests corroborates the findings of a recent systematic review, [6] even though passing an early RTS assessment does seem to correlate with better knee function [30]. In the present study, patients returning to their previous sport had higher Tegner and Lysholm scores than patients who have not returned their previous sport at 2 years.

In the present study, younger age was the only significant predictor of a higher Tegner level at 2 years when co-variates were controlled for. This expands on the recent findings that younger patients have a higher motivation and expectation to return to their previous sport [2, 42]. The difference in preinjury Tegner level and Lysholm score has been previously found as a differing factor between young and old patients undergoing ACLR [38].

The ACL-RSI questionnaire, designed for assessing readiness to return to sport failed to predict any of the outcomes investigated in the present study. It has demonstrated value in younger patients who, overall, have a higher risk of graft injury [23, 24]. In the present study, it was predictive of the 2-year Tegner level on the univariate analysis, but not of the patients’ subjective reported level. In a recent study, ACL-RSI was predictive of a successful RTS, based on the patients’ subjective report, not based on the Tegner questionnaire [18].

In a recent systematic review of 49 studies, only 5 studies reported whether patients were able to successfully return to sport and 90% of studies failed to use objective criteria for permitting returning to sport [13]. A range of hop tests have been suggested as a tool to diagnose lower limb function [31] with studies using a combination thereof [8]. A recent systematic review on the role of hop tests with key outcome variables after an ACL reconstruction found a lack of strong association in 21 studies [22]. A high cutoff for a successful RTS has been demonstrated to reduce the incidence of a reinjury [10], and to predict the level of participation after the injury [41]. In the present study, the cutoff was set comparatively high, and the assessment was performed late, after 9–12 months, giving a high pass rate from the first try. Patients performing better on RTS strength tests at an earlier phase, at 6 months, have been shown to perform better at mid-term follow-up, although they are at a higher risk of a contralateral injury [37]. In the present study, only posterolateral Y balance test was predictive of a higher Tegner level at 2 years, and only on univariate analysis. It combines strength and accuracy of a jump in the posterolateral direction.

By undertaking an RTS assessment and multiple questionnaires, this group of patients are spending considerable time indicating an intent to return to sport. This highly motivated cohort may, therefore, not represent the overall cohort undergoing an ACL reconstruction. The decision not to return to sport is multifactorial, and not only a reflection of the patients’ ability to pass an RTS assessment and these factors were not covered in this study. We have excluded one patient with previous contralateral ACL injuries, which has been shown to be a major factor in changing the expectation for returning to sport [42]. Quads/Hamstrings ratio started to be a part of the RTS assessment in the second half of the study and was thus not used in the study. We did not use a comparison group of patients without a return to sport assessment as their initial motivation was not to return to sport which affects the motivation during rehabilitation significantly, altering all clinical outcomes. Finally, the graft assessment was performed clinically at RTS using the Lachman Test. Pivot shift is used as an assessment tool in our Institute during anaesthesia, prior to the surgical reconstruction [29]. A definite diagnosis of graft rupture can only be performed using MRI.

Surgeons and physiotherapists can utilise the Tegner questionnaire to quickly give the patient an assessment of their likelihood of returning to sport after an ACL reconstruction.

## Conclusion

Preoperative Tegner and expected Tegner level collected prior to an ACL reconstruction can aid in the objective prediction of patients’ return to sport after 2 years. High-level athletes are more likely to return to their previous sport and to the previous level. Younger patients achieve a higher Tegner level at 2 years.

## Electronic supplementary material

Below is the link to the electronic supplementary material.

Supplementary file1 (DOCX 15 kb)
