# Supplementary material for: Tegner level is predictive for successful return to sport 2 years after anterior cruciate ligament reconstruction
Source: Knee Surg Sports Traumatol Arthrosc. 2020 Oct 28;29(9):3010–6. doi: 10.1007/s00167-020-06335-4 (PMC8384787; doi:10.1007/s00167-020-06335-4)
Supplement: Supplementary file 1 — Supplementary file1 (DOCX 15 kb) [file 167_2020_6335_MOESM1_ESM.docx]

Rehabilitation protocol

**Acute (0 - 2 Weeks)**

Goals

Wound healing

Reduce swelling

Regain full extension

Full weight bearing

Wean off crutches

Promote muscle control

Treatment Guidelines

Pain and swelling reduction with ice, intermittent pressure pump, soft tissue massage and exercise

Patella mobilisation

Active range of motion knee exercises, calf and hamstring stretching, contraction (non weight bearing progressing to standing), muscle control and full weight bearing. Aim for full extension by 2 weeks. Full flexion will take longer and generally will come with gradual stretching. Care needs to be taken with hamstring co contraction as this may result in hamstring strains if too vigorous. Light hamstring loading continues into the next stage with progression of general rehabilitation. Resisted hamstring loading should be avoided for approximately 6 weeks

Gait retraining encouraging extension at heel strike

**Stage 2- Quadriceps Control (2-6 Weeks)**

Goals

Full active range of motion

Normal gait with reasonable weight tolerance

Minimal pain and effusion

Develop muscular control for controlled pain free single leg lunge

Avoid hamstring strain

Develop early proprioceptive awareness

Treatment Guidelines

Use active, passive and hands on techniques to promote full range of motion

Progress closed chain exercises (quarter squats and single leg lunge) as pain allows. The emphasis is on pain free loading, VMO and gluteal activation

Introduce gym based exercise equipment including leg press and stationary cycle

Water based exercises can begin once the wound has healed, including treading water, gentle swimming avoiding breaststroke

Begin proprioceptive exercises including single standing leg balance on the ground and mini tramp. This can progress by introducing body movement whilst standing on one leg

Bilateral and single calf raises and stretching

Avoid isolated loading of the hamstrings due to ease of tear. Hamstrings will be progressively loaded through closed chain and gym based activity

**Stage 3- Hamstring/Quadriceps Strengthening (6-12 Weeks)**

Goals

Begin specific hamstring loading

Increase total leg strength

Promote good quadriceps control in lunge and hopping activity in preparation for running

Treatment Guidelines

Focal hamstring loading begins and is progressed steadily throughout the next stages of rehabilitation

Active prone knee flexion which can be quickly progressed to include a light weight and gradually increasing weights

Bilateral bridging off a chair. This can be progressed by moving onto a single leg bridge and then single leg bridge with weight held across the abdomen

Single straight leg dead lift initially active with increasing difficulty by adding dumbbells

With respect to hamstring loading, they should never be pushed into pain and should be carefully progressed. Any subtle strain or tightness following exercises should be managed with a reduction in hamstring based exercises

Gym based activity including leg presses, light squats and stationary bike which can be progressively increased in intensity as pain and control allow. It is important to monitor any effusions following exercise and if it is increasing then exercise should be toned down

Once single leg lunge control is comparable to the other side hopping can be introduced. Hops can be made more difficult by including variations such as forward/back, side to side off a step and in a quadrant

Running may begin towards the latter part of this stage. Prior to running certain criteria must be met:

No anterior knee pain

A pain free lunge and hop that is comparable to the other side

The knee must have no effusion

Before jogging start having brisk walks, ideally on a treadmill to monitor landing action and any effusion. This should be done for several weeks before jogging properly

Increased proprioceptive manoeuvres with standing leg balance and progressive hopping based activity

Expand calf routine to include eccentric loading

**Stage Four-Sport Specific (3-6 Months)**

Goals

Improve leg strength

Develop running endurance speed, change of direction

Advanced proprioception

Prepare for return to sport and recreational lifestyle

Treatment Guidelines

Controlled sport specific activities should be included in the progression of running and gym loads. Increasing effusion post running that isn't easily managed with ice should result in a reduction in running loads

Advanced proprioception to include controlled hopping and turning and balance correction

Monitor potential problems associated with increasing loads

No open chain resisted leg extension exercises unless authorised by Dr Parker

**Stage Five-Return to Sport (6 Months Plus)**

Goals

A safe return to sporting activities

Treatment Guidelines

Full training for 1 month prior to active return to competitive sport

Preparation for body contact sports. Begin with low intensity one on one contests and progress by increasing intensity and complexity in preparation for drills that one might be expected to do at training

To improve running endurance leading up to a normal training session

Full range, no effusion, good quadriceps control for lunge, hopping and hop and turn type activity. Circumference measures of thigh and calf to within 1 cm of other side.
